# Factors Affecting Nursing Students’ Perception of Workplace Bullying

**DOI:** 10.3390/healthcare12161597

**Published:** 2024-08-12

**Authors:** Jeong Sil Choi, Ka Young Kim

**Affiliations:** Department of Nursing, College of Nursing, Gachon University, Incheon 21936, Republic of Korea; jschoi408@empas.com

**Keywords:** bullying, clinical practice, nursing education, nursing student, workplace bullying

## Abstract

Workplace bullying is a critical and prevalent issue that causes serious problems in healthcare settings. However, there is little research on the factors affecting nursing students’ perception of workplace bullying despite their forthcoming transition into the nursing profession. Therefore, this study aimed to identify the factors related to nursing students’ perception of workplace bullying in Korea. A cross-sectional study was conducted among 242 nursing students who had experienced clinical practice. The survey questionnaire included general characteristics, perceived susceptibility and severity of bullying, and perception of workplace bullying. Data were analyzed using multiple regression analysis. In this study, the significant factors affecting nursing students’ perception of workplace bullying included bullying experience in clinical practice and the perceived severity of bullying. Therefore, it is crucial for nursing managers and instructors to have a clear understanding of the bullying situations experienced by nursing students during clinical practice. We should promote the perception of workplace bullying through indirect experiences such as systematic education about workplace bullying for nursing students, which may prevent workplace bullying in clinical practice and work environments. Furthermore, a comprehensive and multifaceted approach is necessary to effectively prevent workplace bullying in clinical practice and work environments. This study reveals that systemic and persistent education and intervention to bullying may improve nursing students’ perception of workplace bullying and prevent workplace bullying in clinical practice and work environments. Furthermore, this study provides basic data on the prevention and management of bullying in nursing students’ clinical practice.

## 1. Introduction

University education provides new information and skills essential for future jobs and enhancing learning and research [1]. It is essential to build a positive job image, particularly in nursing education, including clinical practice. Clinical practice enables nursing students to apply theoretical knowledge in clinical settings and perform actual nursing care [2,3,4]. Furthermore, it allows them to experience their role as a future nurse in advance. However, many nursing students experience anxiety and stress in clinical practice [5,6]. Particularly, stress and anxiety are common in the first clinical practice and training [7,8]. The exacerbation and maintenance of these psychological symptoms can cause several problems in nursing students [9]. A previous study reported that stress resulted in psychological problems, physical distress, behavioral problems, and poor academic achievement [3]. Stress and anxiety were associated with reduced coping behaviors such as problem solving, self-criticism, wishful thinking, social support, cognitive restructuring, and social withdrawal in nursing students [9]. Recently, bullying has been reported as a major stressor in clinical practice [10,11].

Bullying has emerged as a major concern in clinical education and healthcare workplaces [11,12]. It means harassing, repeated, and targeted behaviors that humiliate and cause distress in the recipient [13,14]. Particularly, workplace bullying is prevalent in many workplaces worldwide, despite having national and organizational culture diversity [14,15,16,17]. According to the 2017 national survey on workplace bullying institutes, approximately 20% of the workers reported experiencing workplace bullying in the United States [18].

Workplace bullying in healthcare settings, including hospitals, is a critical and prevalent issue that causes serious problems. A systematic review reported that workplace bullying had a pooled prevalence of 22.2% among Korean nurses [19]. A Polish study survey found that about 66% of nurses experienced workplace bullying [20]. Most workplace bullying in nursing is reported to threaten the nursing profession as a whole as well as the safety of patients and nurses despite the diversity of prevalence [21,22,23]. It is a prevalent psychosocial risk factor in the workplace [15,24,25]. Workplace bullying results in health problems, including psychological stress and physical health, and it is closely associated with the intention to leave the profession [14,15,20]. Particularly, the nursing profession is vulnerable to workplace bullying due to hierarchical and departmental organizational structures [26,27]. Reportedly, the factors affecting workplace bullying include age, seniority, working hours per week, and bachelor’s degree [20]. Other significant factors affecting workplace bullying include the career in the present department, educational experience to prevent violence in the workplace, relation-oriented culture, and hierarchy-oriented culture [28]. The perception of workplace bullying and the factors affecting it are essential issues for nursing students as well as nurses. Workplace bullying in nursing reportedly begins in the nursing university and continues in the nursing profession [21]. In particular, the obscure fear of workplace bullying during clinical practice not only decreases the achievement of clinical learning but may also affect their choice of nursing as a career [29,30].

Therefore, this study investigated the factors affecting nursing students’ perception of workplace bullying in Korea. Furthermore, this study provides basic data on the prevention and management of bullying in nursing students’ clinical practice.

## 2. Materials and Methods

### 2.1. Study Design and Participants

We performed a cross-sectional study to identify the factors affecting nursing students’ perception of workplace bullying in Korea. The sample size using G*Power 3.1.9.2 was calculated as 172 for linear multiple regression analysis based on an effect size of 0.15, significance level of 0.05, power of 0.95, and 10 predictors [31]. About 250 questionnaires were circulated after considering the probability of dropping out. Of these, 245 were returned. Finally, responses from 242 participants were analyzed after excluding 3 participants due to insufficient responses, resulting in a 96.8% response rate. Ethical approval was obtained from the Institutional Review Board of the Gachon University (IRB No. 1044396-201910-HR-183-01). Written informed consent was obtained from all the participants.

### 2.2. Data Collection

Data were collected from third- and fourth-grade nursing students who had experienced clinical practice in the hospital of the metropolitan area of Korea from 1 December to 30 December 2019. The survey items included general characteristics, perceived susceptibility of bullying, perceived severity of bullying, and perception of workplace bullying. This survey was conducted as a self-administered questionnaire after a researcher who did not attend clinical practice explained the study’s purpose and background.

### 2.3. Variables and Measures

Participants’ sociodemographic characteristics included age, sex, year in school, the presence of a notice related to bullying in the university, the presence of a notice related to bullying in the hospital, and the presence of bullying experience in clinical practice. The items on the perceived susceptibility and severity of bullying were developed based on research by Park and Lee [32]. This scale contained two items on the perceived susceptibility and severity of bullying. The items were rated on a five-point Likert scale: (1) strongly disagree, (2) disagree, (3) neither agree nor disagree, (4) agree, and (5) strongly agree. The content validity index (CVI) of perceived susceptibility and severity were 0.95 and 0.95, respectively. The reliability using Cronbach’s alpha for perceived susceptibility and severity was 0.76 and 0.96, respectively. Furthermore, items on the perception of workplace bullying were developed based on the research by Haq [33]. It contained six items under five subscales: one item on meaning, one item on target, one item on cause, two items on effect, and one item on coping. The items were rated on a five-point Likert scale: (1) strongly disagree, (2) disagree, (3) neither agree nor disagree, (4) agree, and (5) strongly agree. The content validity of the developed items was verified by four experts. The reliability using Cronbach’s alpha in the pilot study and this study was 0.89 and 0.91, respectively.

### 2.4. Data Analysis

All statistical analyses were performed using IBM SPSS Statistics 25.0 software (IBM Corp., Armonk, NY, USA). The data were expressed as frequencies, percentages, means, and standard deviations. Linear multiple regression was performed to determine factors affecting nursing students’ perception of workplace bullying. Significance was defined as *p* < 0.05.

## 3. Results

### 3.1. Differences in the Perception of Workplace Bullying by Participants’ Characteristics

Differences in the total and detailed items (meaning, target, cause, effect, coping) of the perception of workplace bullying according to participants’ characteristics are shown in Table 1. This study included 242 nursing students who had experienced clinical practice. The average age was 21.73 years, 205 (84.7%) participants were female, and 122 (50.4%) were 4th year students. About 60 (24.8%) and 20 (8.3%) participants had taken the notice related to bullying in the university and hospital, respectively. About 89 (36.8%) had experienced bullying in clinical practice. Furthermore, the perception of workplace bullying shows a significant difference according to the bullying experience in clinical practice (*p* = 0.024). The perception of workplace bullying differed significantly according to sex (*p* = 0.016), year in school (*p* = 0.007), and bullying experience in clinical practice (*p* = 0.013) in the cause subscale, bullying experience in clinical practice (*p* = 0.049) in the effect subscale, and notice related to bullying in the hospital (*p* = 0.041) in the coping subscale.

### 3.2. Levels of Perceived Susceptibility of Bullying, Perceived Severity of Bullying, and Perception of Workplace Bullying among Nursing Students

Table 2 shows questions and scores for the perceived susceptibility of bullying, perceived severity of bullying, and perception of workplace bullying among nursing students who had experienced clinical practice. The total mean answer scores related to the susceptibility and severity of bullying were 2.66 (SD = ±1.08) and 3.67 (SD = ±1.27) out of 5, respectively. The detailed questions related to the perceived susceptibility of bullying were “I seem to be harassed more easily than others (2.15 ± 1.05)” and “I am afraid of being bullied (3.17 ± 1.34)”. The questions related to the perceived severity of bullying were “I think being bullied will have a serious impact on me (3.69 ± 1.30)” and “I think being bullied will have an impact on my future (3.64 ± 1.28)”.

The total mean score related to nursing students’ perception of workplace bullying was 3.99 (SD = ±0.48) out of 5. The perception of workplace bullying consisted of the meaning, target, cause, effect, and coping with workplace bullying. The mean scores of the meaning, target, cause, effect, and coping with workplace bullying among nursing students were “It is important to know what bullying in the workplace is (3.83 ± 0.85)”, “I think that the target of bullying in the workplace is mainly a new nurse (4.00 ± 0.85)”, “I think workplace bullying is affected by organizational culture or hospital environment (4.58 ± 0.62)”, “I think workplace bullying can lead to anxiety and depression (4.52 ± 0.67) and I think workplace bullying can lead to poor mental and physical health (4.45 ± 0.73)”, and “I know how to deal with bullying in the workplace (2.58 ± 1.07)”, respectively. The meaning, target, cause, and coping with workplace bullying included one question each, while the effect of workplace bullying included two questions.

### 3.3. Correlations among the Study Variables

Table 3 shows the correlations between perceived susceptibility and severity of bullying and the perception of workplace bullying among nursing students. Statistically significant correlations were found between the perception of workplace bullying and the perceived severity of bullying (*p* = 0.001). The subscales of the perception of workplace bullying, including meaning (*p* = 0.019), target (*p* = 0.001), cause (*p* < 0.001), effect (*p* = 0.007), and coping (*p* = 0.004), showed significant correlations with the perceived severity of bullying.

### 3.4. Factors Affecting Nursing Students’ Perception of Workplace Bullying

The multiple regression analysis revealed that the significant factors affecting nursing students’ perception of working bullying were bullying experience in clinical practice (β = 0.136, *p* = 0.036) and the perceived severity of bullying (β = 0.279, *p* = 0.001) (Table 4).

## 4. Discussion

This study demonstrated the factors affecting nursing students’ perception of workplace bullying in Korea. The significant factors affecting nursing students’ perception of workplace bullying were bullying experience in clinical practice and the perceived severity of bullying.

There are few studies and research tools on nursing students’ perception of workplace bullying despite the prevalence and severity of workplace bullying in the nursing profession [14,15]. Workplace bullying in nursing is a pervasive and serious problem in the work environment [21,25]. However, it is a critical issue that can directly and indirectly affect nursing students and healthcare workers, including nurses. Reportedly, workplace bullying in nursing begins in the nursing university and continues in the nursing profession [21]. Therefore, we focused on the perception of workplace bullying among nursing students. Furthermore, this study has suggested a simple research tool that includes the meaning, target, cause, effect, and coping to investigate the perception of workplace bullying among nursing students [33]. The results revealed the lowest score on the question of whether or not to cope with workplace bullying. Even nurses are vulnerable in coping with workplace bullying, and several studies have focused on coping strategies of workplace bullying and work stress [34,35]. Understanding and investigating the factors affecting nursing students’ perception of workplace bullying is helpful in preventing bullying in practical education and may be positively affected to recognize and prevent workplace bullying as a nurse in the near future.

The results of this study have important implications in nursing education. Since there is a close association between perception and coping skills, an accurate perception and understanding of workplace bullying can enable appropriate coping with workplace bullying among nursing students as preliminary nurses in the present clinical practice and the future workplace [36]. In this study, bullying experience in clinical practice affected nursing students’ perception of workplace bullying. The bullying experience in clinical practice may cause psychological stress, reduced academic performance, and withdrawal from social contacts in the current university life [3,37]. Furthermore, in the near future workplace, it may be followed by persistent psychological problems, social anxiety and withdrawal, and intention to leave the workplace [14,15,17,20]. Therefore, it is crucial for nursing managers and instructors to have clear a understanding of the bullying situations experienced by nursing students during clinical practice. Similar to the experience of bullying that affects the perception of workplace bullying, we should promote the perception of workplace bullying through indirect experiences such as systematic education about workplace bullying for nursing students, which may prevent workplace bullying in clinical practice and work environments.

Moreover, the perceived severity of bullying affected nursing students’ perception of workplace bullying. Perceived severity and perceived susceptibility are measuring constructs of the health belief model and are widely used in determining and predicting health behaviors [38]. Particularly, perceived severity represents the subjective perception of the risk of having a disease or harmful condition as a result of a particular behavior [39]. Perceived severity of bullying affected nursing students’ perception of workplace bullying in this study. An increase in the perceived risk increases precautionary health-related behaviors [40]. Thus, perceived severity of bullying affects the perception of workplace bullying and may ultimately prevent bullying in the clinical practice and work environment. Nursing education should develop programs and provide systemic and persistent education and interventions to prevent workplace bullying.

A comprehensive and multifaceted approach is necessary to effectively prevent workplace bullying in clinical practice and work environments. To develop an educational program to enhance perception of workplace bullying, meaning, target, cause, effect, and coping can be included. Such programs should be implemented for all members within university and hospital, including nursing students, instructors, nurses, and healthcare professionals. A policy-based approach is essential for preventing workplace bullying within institutions. This includes implementing a structured system for reporting bullying incidents, establishing counseling services and support groups for victims and witnesses, and ensuring regular monitoring and evaluation, all of which should be easily accessible to members [41]. Furthermore, it is important to set standards for appropriate workplace behavior through leadership and role modeling by leaders and managers [42].

However, this study has some limitations. First, while significant factors affecting nursing students’ perception of workplace bullying are described, the explanatory power was low at 5.7%. Therefore, further research is needed to explore additional variables that may impact nursing students’ perception of workplace bullying. Second, since workplace bullying is diverse depending on the national and organizational culture, frequent studies concerning various national and organizational cultures are required. Third, although the clinical practice environment does not change abruptly, it is important to account for the timing of data collection. Furthermore, it is necessary to consider the timing of the study when applying the finding to the current situation.

## 5. Conclusions

This study demonstrated the factors affecting nursing students’ perception of workplace bullying. Bullying experience in clinical practice and perceived severity of bullying affected nursing students’ perception of workplace bullying. Further research and education programs are required in nursing education to prevent and manage workplace bullying. This study reveals that systemic and persistent education and intervention to bullying may improve nursing students’ perception of workplace bullying and prevent workplace bullying in clinical practice and work environments. Furthermore, this study provides basic data on the prevention and management of bullying in nursing students’ clinical practice.

## Figures and Tables

**Table 1:** Differences in the perception of workplace bullying by participants’ characteristics.

Variables		Total			Detailed Items (M ± SD)			
		N (%)	M ± SD	Meaning	Target	Cause	Effect	Coping
Sex	Male	37 (15.3)	3.94 ± 0.60	3.86 ± 1.00	3.84 ± 0.99	4.15 ± 0.93	4.46 ± 0.73	3.16 ± 1.14
	Female	205 (84.7)	4.00 ± 0.46	3.82 ± 0.82	4.03 ± 0.82	4.55 ± 0.58	4.60 ± 0.59	2.48 ± 1.02
	*p*		0.527	0.766	0.205	0.016	0.201	<0.001
Year in school	3rd	120 (49.6)	3.96 ± 0.53	3.77 ± 0.88	4.01 ± 0.82	4.37 ± 0.76	4.59 ± 0.67	2.67 ± 1.08
	4th	122 (50.4)	4.02 ± 0.43	3.89 ± 0.83	3.99 ± 0.88	4.60 ± 0.53	4.57 ± 0.56	2.50 ± 1.05
	*p*		0.329	0.280	0.879	0.008	0.742	0.226
Notice related to bullying in the university	No	182 (75.2)	3.98 ± 0.49	3.80 ± 0.86	4.01 ± 0.86	4.48 ± 0.68	4.60 ± 0.62	2.52 ± 1.09
	Yes	60 (24.8)	4.03 ± 0.46	3.90 ± 0.82	3.98 ± 0.79	4.52 ± 0.60	4.50 ± 0.60	2.78 ± 0.99
	*p*		0.457	0.441	0.861	0.675	0.255	0.093
Notice related to bullying in the hospital	No	222 (91.7)	3.98 ± 0.48	3.82 ± 0.86	3.97 ± 0.86	4.48 ± 0.67	4.58 ± 0.62	2.54 ± 1.07
	Yes	20 (8.3)	4.18 ± 0.46	3.95 ± 0.83	4.30 ± 0.57	4.60 ± 0.62	4.60 ± 0.60	3.05 ± 0.95
	*p*		0.065	0.499	0.097	0.421	0.871	0.041
Bullying experience in clinical practice	No	153 (63.2)	3.94 ± 0.49	3.78 ± 0.86	3.92 ± 0.91	4.41 ± 0.72	4.52 ± 0.66	2.61 ± 1.02
	Yes	89 (36.8)	4.08 ± 0.45	3.91 ± 0.83	4.13 ± 0.69	4.62 ± 0.52	4.67 ± 0.52	2.54 ± 1.16
	*p*		0.024	0.244	0.058	0.013	0.049	0.631

**Table 2:** Levels of perceived susceptibility of bullying, perceived severity of bullying, and perception of workplace bullying among nursing students.

Items	Mean ± SD
Perceived susceptibility of bullying (1~5)	2.66 ± 1.08
I seem to be harassed more easily than others.	2.15 ± 1.05
I am afraid of being bullied.	3.17 ± 1.34
Perceived severity of bullying (1~5)	3.67 ± 1.27
I think being bullied will have a serious impact on me.	3.69 ± 1.30
I think being bullied will have an impact on my future.	3.64 ± 1.28
Perception of workplace bullying (1~5)	3.99 ± 0.48
Meaning of workplace bullying	3.83 ± 0.85
It is important to know what bullying in the workplace is.	
Target of workplace bullying	4.00 ± 0.85
I think that the target of bullying in the workplace is mainly a new nurse.	
Cause of workplace bullying	4.58 ± 0.62
I think workplace bullying is affected by organizational culture or hospital environment.	
Effect of workplace bullying	4.49 ± 0.66
I think workplace bullying can lead to anxiety and depression.	4.52 ± 0.67
I think workplace bullying can lead to poor mental and physical health.	4.45 ± 0.73
Coping with workplace bullying	2.58 ± 1.07
I know how to deal with bullying in the workplace.	

**Table 3:** Correlations between the perceived susceptibility and severity of bullying and the perception of workplace bullying among nursing students.

	Perception of Workplace Bullying (r/(*p*))					
	Total	Meaning	Target	Cause	Effect	Coping
Perceived susceptibility of bullying	0.082 (0.204)	0.015 (0.815)	0.212 (0.001)	0.230 (<0.001)	0.110 (0.087)	−0.306 (<0.001)
Perceived severity of bullying	0.206 (0.001)	0.150 (0.019)	0.219 (0.001)	0.279 (<0.001)	0.174 (0.007)	−0.183 (0.004)

**Table 4:** Factors affecting nursing students’ perception of workplace bullying.

Variables	β	t (*p*)	R^2^	Adj. R^2^	F
Constant	0.268	13.428 (<0.001)	0.080	0.057	3.418 **
Sex	−0.010	−0.168 (0.867)			
Year in school	0.035	0.556 (0.579)			
Notice related to bullying in the hospital	0.100	1.576 (0.116)			
Bullying experience in clinical practice	0.136	2.105 (0.036)			
Perceived susceptibility of bullying	−0.125	−1.471 (0.143)			
Perceived severity of bullying	0.279	3.285 (0.001)			

** *p* < 0.01.

## Data Availability

The data that support the findings of this study are available on request from the corresponding author.
